# The long non-coding RNA *EPB41L4A-AS2* inhibits tumor proliferation and is associated with favorable prognoses in breast cancer and other solid tumors

**DOI:** 10.18632/oncotarget.8007

**Published:** 2016-03-09

**Authors:** Shouping Xu, Peiyuan Wang, Zilong You, Hongxue Meng, Guannan Mu, Xianan Bai, Guangwen Zhang, Jinfeng Zhang, Da Pang

**Affiliations:** ^1^ Department of Breast Surgery, Harbin Medical University Cancer Hospital, Harbin, China; ^2^ Department of Pathology, Harbin Medical University Cancer Hospital, Harbin, China; ^3^ Biotherapy Center, Harbin Medical University Cancer Hospital, Harbin, China; ^4^ Heilongjiang Academy of Medical Sciences, Harbin, China

**Keywords:** antisense lncRNA, EPB41L4A-AS2, prognostic value, proliferation, breast cancer

## Abstract

*EPB41L4A-AS2* is a novel long non-coding RNA of unknown function. In this study, we investigated the expression of *EPB41L4A-AS2* in breast cancer tissues and evaluated its relationship with the clinicopathological features and prognosis of patients with breast cancer. This entailed conducting a meta-analysis and prognosis validation study using two cohorts from the Gene Expression Omnibus (GEO). In addition, we assessed *EPB41L4A-AS2* expression and its relationship with the clinicopathological features of renal and lung cancers using the Cancer Genome Atlas cohort and a GEO dataset. We also clarified the role of *EPB41L4A-AS2* expression in mediating cancer cell proliferation in breast, renal, and lung cancer cell lines transfected with an *EPB41L4A-AS2* expression vector. We found that high *EPB41L4A-AS2* expression is associated with favorable disease outcomes. Gene ontology enrichment analysis revealed that *EPB41L4A-AS2* may be involved in processes associated with tumor biology. Finally, overexpression of *EPB41L4A-AS2* inhibited tumor cell proliferation in breast, renal, and lung cancer cell lines. Our clinical and *in vitro* results suggest that *EPB41L4A-AS2* inhibits solid tumor formation and that evaluation of this long non-coding RNA may have prognostic value in the clinical management of such malignancies.

## INTRODUCTION

Up to 30% of node-negative and 70% of node-positive breast cancer patients develop distant metastases during the course of their illness [1, 2]. Once distant metastases occur, breast cancer is no longer considered curable [3]. Despite a large amount of information on breast cancer risk factors, it is not understood how these risks influence metastases [4, 5]. Known risk factors account for only a small proportion of the overall incidence in high-risk populations; therefore, investigations into other risk factors may reduce breast cancer morbidity and mortality while providing high-quality screening, diagnosis, and treatment for patients who are at risk for developing breast cancer.

Long non-coding RNAs (lncRNA) are transcripts longer than 200 nucleotides with little or no protein-coding capacity [6]. LncRNAs regulate gene expression at various levels, including chromatin modifications, transcription, and post-transcriptional processing [7–11]. Recent studies have demonstrated that lncRNAs are involved in tumorigenesis and can be used as cancer biomarkers [12–21]. The lncRNAs *PCA3* and *UCA1* were identified as sensitive, specific, and unique diagnostic markers for human prostate cancer and bladder cancer, respectively [22–24]. In addition, *SChLAP1* is an lncRNA biomarker associated with metastatic progression in prostate cancer [20]. The lncRNA, *BCAR4*, induces antiestrogen resistance in breast cancer, whereas *PVT1* and *MRUL* induce multidrug-resistance in gastric cancer [25–28]. Moreover, several lncRNAs influence prognosis in solid tumors, such as *GAPLINC* in gastric cancer, *UBC1* and *GAS5* in bladder cancer, *HOTTIP* in hepatocellular carcinoma, and *HOTAIR* in breast cancer and colorectal cancer [29–36]. For single-gene disorders, a therapy for Angelman syndrome has been successfully performed by targeting an lncRNA *UBE3A-ATS* [37].

In searching approximately 100 breast cancer microarray datasets on the NCBI Gene Expression Omnibus (GEO) website, we found that a novel lncRNA, *EPB41L4A-AS2*, was closely associated with several vital tumor pathophysiological processes including tumorigenesis, chemoresistance, and estrogen regulation (http://www.ncbi.nlm.nih.gov/geoprofiles). *EPB41L4A-AS2* is located in the 5q22.2 region of the genome and is an antisense lncRNA [38]. There is nothing in the literature about the biological function or association of *EPB41L4A-AS2* with cancer or other diseases. To investigate the clinical relevance of *EPB41L4A-AS2* and its biological role in breast cancer, we measured the expression of *EPB41L4A-AS2* and its association with clinical and pathological features in 250 breast tumor samples, and further validated our findings using The Cancer Genome Atlas (TCGA) dataset. Next, we examined the association of *EPB41L4A-AS2* expression with survival of different solid tumor patients in our breast cancer patient cohort along with two other GEO cohorts (renal cancer and lung cancer). To validate the prognostic value of *EPB41L4A-AS2* expression and confirm our clinical findings, we conducted a meta-analysis in a fourth GEO cohort with 3699 breast cancer patients. We also performed gene ontology term enrichment analysis, and found that *EPB41L4A-AS2* is involved with many tumor-associated biological processes. Finally, we showed that overexpression of *EPB41L4A-AS2* inhibits tumor cell growth in breast cancer, renal cancer, and lung cancer cell lines. A flowchart of this study is shown in Supplementary Figure S1. Overall, our findings indicate that *EPB41L4A-AS2* may be a prognostic biomarker and can act as a tumor suppressor in mediating the proliferation of solid tumors.

## RESULTS

### Low *EPB41L4A-AS2* expression is correlated with adverse clinicopathological features in breast cancer

**Figure 1:**
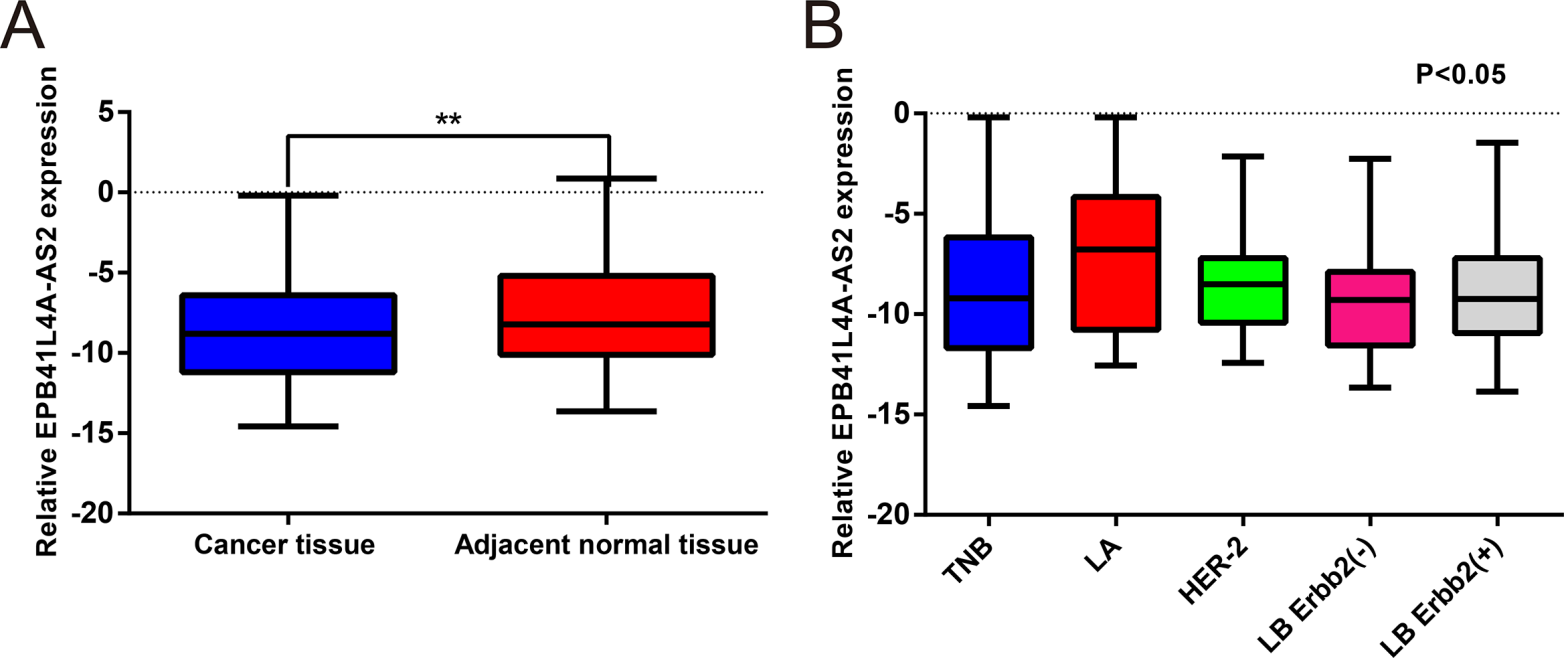
*EPB41L4A-AS2* expression in human breast cancer tissues and adjacent non-cancerous tissues by qRT-PCR (**A**) Relative expression of *EPB41L4A-AS2* in 250 breast cancer tissues and 50 adjacent non-cancerous tissues. (**B**) Relative expression of *EPB41L4A-AS2* among five breast cancer molecular subtypes. qRT-PCR amplification was performed in triplicate and the levels of *EPB41L4A-AS2* were normalized against control GAPDH expression. LA: Luminal A subtype; LB: Luminal B subtype; TNB: Triple negative subtype; ****p < 0.0001; ***p < 0.001; **p < 0. 01; *p < 0.05.

To assess the expression of *EPB41L4A-AS2* in breast cancer, 250 breast cancer tissues and 50 adjacent normal tissues (Cohort I) were examined by quantitative real-time PCR (qRT-PCR). Expression of *EPB41L4A-AS2* in breast cancer tissues was categorized as high or low according to the median value. *EPB41L4A-AS2* levels were downregulated in breast cancer tissues compared with corresponding normal tissues (*p* = 0.005; Figure 1A). Among five breast cancer molecular subtypes, *EPB41L4A-AS2* was more highly expressed in the luminal A subtype than the other four subtypes (*p* < 0.001; Figure 1B). We then investigated the relationship between *EPB41L4A-AS2* expression and clinicopathological characteristics in our cohort. Larger tumor size (*p* = 0.026), higher stage and grade (*p* = 0.016 and 0.014, respectively), and ER and PR negative expression (*p* = 0.013 and 0.021, respectively) were correlated with low *EPB41L4A-AS2* expression. However, *EPB41L4A-AS2* expression was not associated with age, HER-2 status, or Ki67 status (*p* > 0.05; Table 1).

**Table 1:** Associations of *EPB41L4A-AS2* expression with clinicpathological factors

Patient Features	Total No. (%)	Low *EPB41L4A-AS2* No. (%)	High *EPB41L4A-AS2* No. (%)	*P* value
**Age (years)**	250			0.357
< 50	160 (64.00)	84 (52.50)	76 (47.50)	
≥ 50	90 (36.00)	41 (45.56)	49 (54.44)	
**Tumor size**	250			0.026
T1	65 (26.00)	24 (36.92)	41 (63.08)	
T2	138 (55.20)	72 (52.17)	66 (47.83)	
T3	47 (18.80)	29 (61.70)	18 (38.30)	
**Tumor Stage**	250			0.016
Stage I	42 (16.80)	14 (33.33)	28 (66.67)	
Stage II	168 (67.20)	85 (50.60)	83 (49.40)	
Stage III	40 (16.00)	26 (65.00)	14 (35.00)	
**Tumor Grade**	250			0.034
Grade 1	44 (17.60)	16 (36.36)	28 (63.64)	
Grade 2	102 (40.80)	48 (47.06)	54 (52.94)	
Grade 3	104 (41.60)	61 (58.65)	43 (41.35)	
**ER status**	250			0.013
Positive	156 (62.40)	68 (43.59)	88 (56.41)	
Negative	94 (37.60)	57 (60.64)	37 (39.36)	
**PR status**	250			0.021
Positive	147 (58.80)	64 (43.54)	83 (56.46)	
Negative	103 (42.20)	61 (59.22)	42 (41.78)	
**Her-2 status**	250			0.871
Positive	46 (18.40)	24 (52.17)	22 (47.83)	
Negative	204 (72.60)	101 (49.51)	103 (50.49)	
**Ki67 status**	250			0.591
< 14%	83 (33.20)	39 (46.99)	44 (63.01)	
≥ 14%	167 (67.80)	86 (51.50)	81 (48.50)	

### *EPB41L4A-AS2* expression is reduced in several malignant tumors

**Figure 2:**
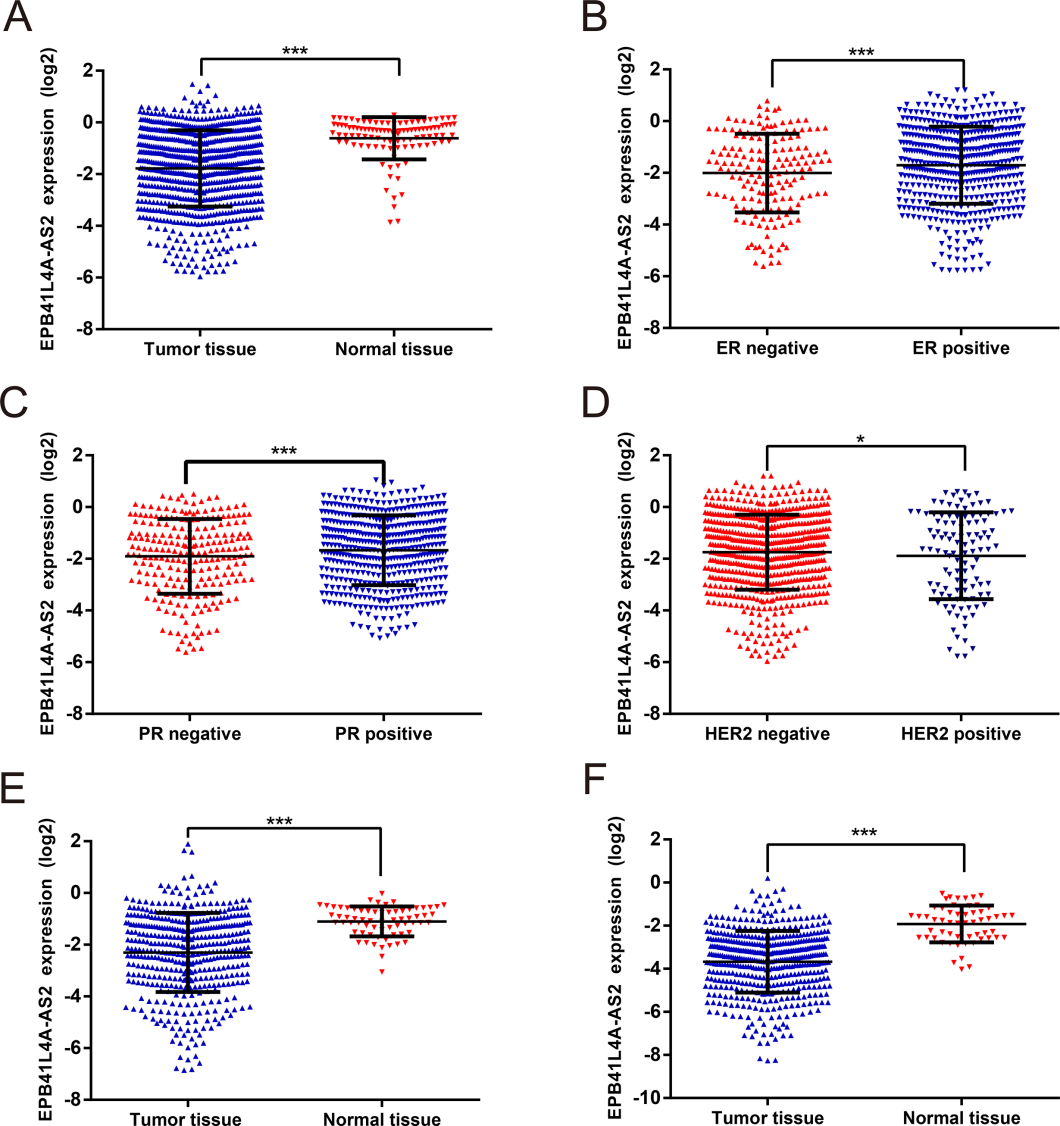
*EPB41L4A-AS2* expression in different solid tumors in TCGA *EPB41L4A-AS2* expression: (**A**) in breast cancer tissues (*N* = 837) and normal tissues (*N* = 105), (**B**) in breast cancer with ER positive (*N* = 584) or negative (*N* = 174), (**C**) in breast cancer with PR positive (*N* = 508) or negative (*N* = 247), (**D**) in breast cancer with HER-2 positive (*N* = 113) or negative (*N* = 630), (**E**) in renal cancer tissues (*N* = 448) and normal tissues (*N* = 47), (**F**) in lung cancer tissues (*N* = 478) and normal tissues (*N* = 58). ***p < 0. 001; *p < 0.05.

We next utilized data from the TCGA to determine whether the expression of *EPB41L4A-AS2* was similar in a large cohort of patients with different solid tumors (Cohort II, https://tcga-data.nci.nih.gov/). In Cohort II, *EPB41L4A-AS2* levels were again downregulated in breast cancer tissues compared with the corresponding normal tissues (*p* < 0.001; Figure 2A). Patients with positive hormone receptor expression had higher *EPB41L4A-AS2* levels than those with negative receptor status (*p* < 0.001 for ER or PR; Figure 2B–2C). In addition, higher *EPB41L4A-AS2* expression was observed in patients with HER-2 negative status compared with those with positive status (*p* = 0.036; Figure 2D). In the PAM50 molecular subtype system, higher *EPB41L4A-AS2* expression was observed in patients with the Luminal A subtype compared with other subtypes (*p* < 0.05; Supplementary Figure S2).

In addition to breast cancer, we also analyzed other solid tumors such as renal cancer and lung cancer. As expected, low expression of *EPB41L4A-AS2* was observed in renal cancer tissues compared with normal tissues (*p* < 0.001; Figure 2E). Expression of *EPB41L4A-AS2* in tumors with high tumor stage or grade was also downregulated compared with those with low tumor stage or grade (*p* < 0.05; Supplementary Figures S3 and S4). We also saw similar results in lung cancer tissues vs. normal tissues (*p* < 0.001; Figure 2F). Thus, tumors with more malignant characteristics have lower levels of *EPB41L4A-AS2*, suggesting that *EPB41L4A-AS2* may be a tumor suppressor.

### High *EPB41L4A-AS2* expression is associated with better overall survival of breast cancer patients

**Figure 3:**
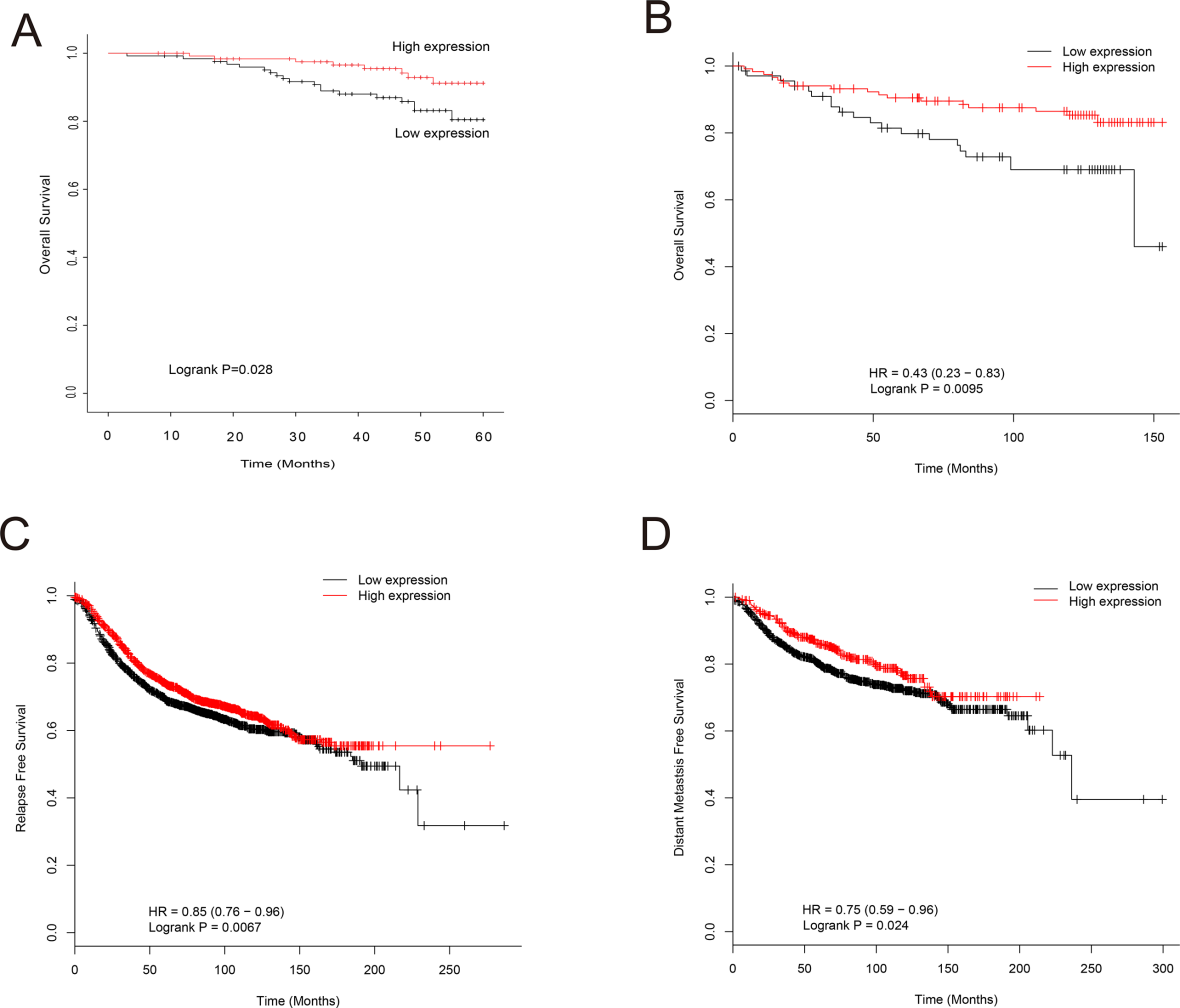
Association of *EPB41L4A-AS2* expression with survival of breast cancer patients (**A**) *EPB41L4A-AS2* expression and OS of breast cancer patients in cohort I (*N* = 250). (**B**) *EPB41L4A-AS2* expression and OS in TP53 wild-type patients in cohort III (*N* = 187). (**C**) *EPB41L4A-AS2* expression and RFS of breast cancer patients in cohort III (*N* = 3,554). (**D**) *EPB41L4A-AS2* expression and DMFS of breast cancer patients in cohort III (*N* = 1,609).

The relationship between *EPB41L4A-AS2* expression and prognosis was investigated using both cohorts I and III (from the GEO database). In cohort I, breast cancer patients with low *EPB41L4A-AS2* expression had poor overall survival (OS) when compared with patients with high expression (*p* = 0.028; Figure 3A). This result was validated in cohort III, which consisted of 26 breast cancer datasets containing clinicopathologic information (Supplementary Table S1). High *EPB41L4A-AS2* expression was associated with better OS than low expression in patients with ER positive breast cancer, low tumor grade, or uninvolved lymph nodes (all *p* < 0.05; Supplementary Figures S5–S7).

*TP53* mutations are common in breast cancers and typically lead to chemoresistance. We next explored the relationship between *TP53* mutations and *EPB41L4A-AS2* expression in breast cancer patients. A better OS was observed in *TP53* wild-type breast cancer patients with high *EPB41L4A-AS2* expression, while the converse was found for breast cancer patients with a *TP53* mutation (*p* < 0.01 and *p* = 0.43, respectively; Figure 3B and Supplementary Figure S8). For relapse-free survival (RFS) and distant metastasis-free survival (DMFS), breast cancer patients with high *EPB41L4A-AS2* expression had better prognoses than those with low levels of *EPB41L4A-AS2* (*p* < 0.01, *p* = 0.024, respectively; Figure 3C–3D). A meta-analysis was also performed to further validate the above results in a larger population (Cohort IV) of 24 datasets in GEO with 3699 breast cancer patients (Supplementary Table 2). We again found that high *EPB41L4A-AS2* expression results in a better prognosis for any event of relapse, metastasis, or death in breast cancer patients (*p* = 0.0175; Supplement Figure S9).

### High *EPB41L4A-AS2* expression is associated with better overall survival of renal and lung cancer patients

**Figure 4:**
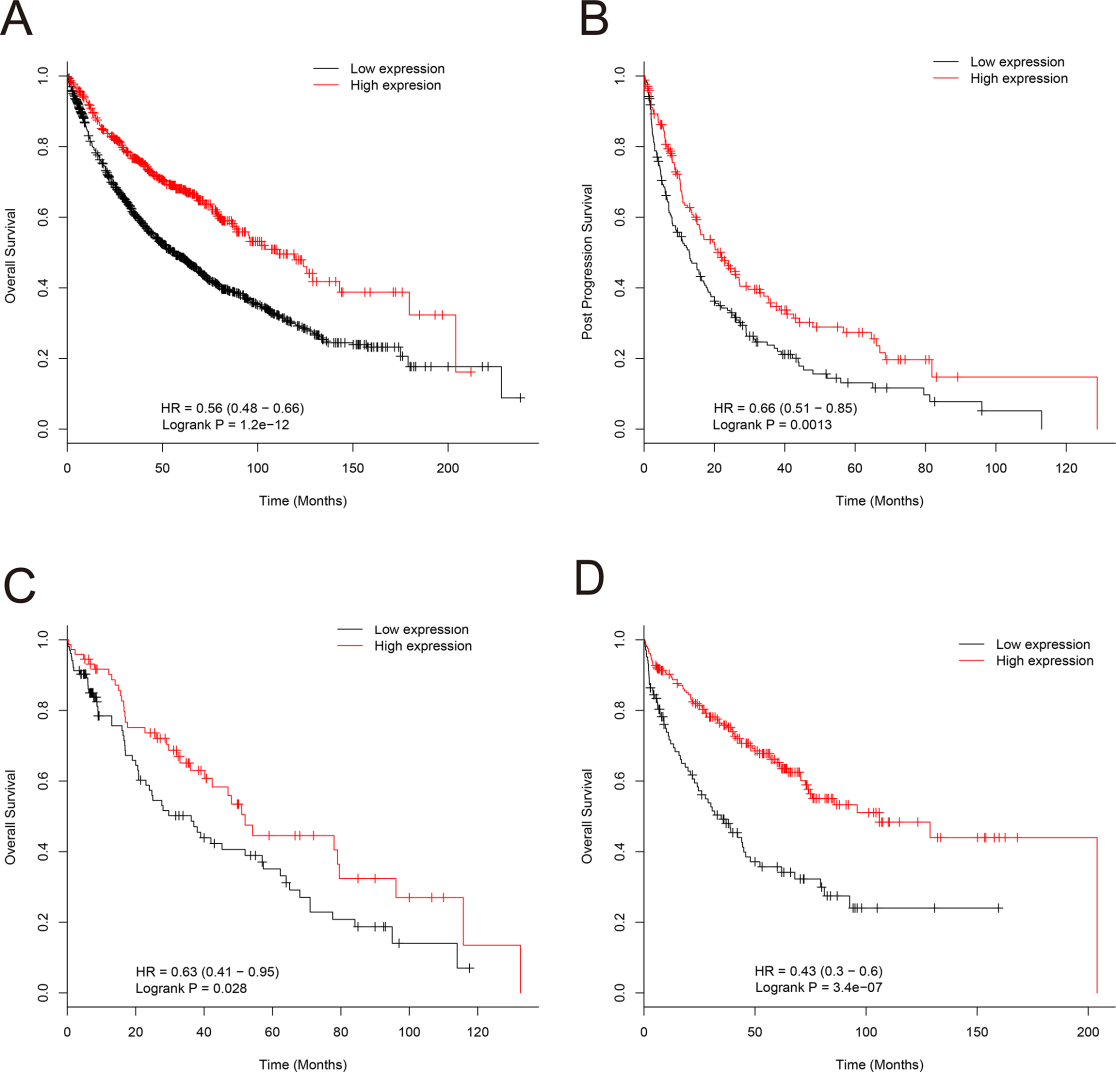
Association of *EPB41L4A-AS2* expression with lung cancer patient survival in cohort III (**A**) *EPB41L4A-AS2* expression and OS of lung cancer patients (*N* = 1926). (**B**) *EPB41L4A-AS2* expression and post-progression survival of lung cancer patients (*N* = 344). (**C**) *EPB41L4A-AS2* expression and OS of lung cancer patients with chemotherapy (*N* = 176), and without chemotherapy (*N* = 310) (**D**).

We also examined the relationship between *EPB41L4A-AS2* expression and prognosis in cohorts II and III with renal cancer and lung cancer. In cohort II, renal cancer patients with high *EPB41L4A-AS2* expression had better OS than those with low *EPB41L4A-AS2* expression, which was consistent with our findings in breast cancer patients (*p* < 0.001, Supplementary Figure S10). In cohort III, lung cancer patients with high *EPB41L4A-AS2* expression also had better OS and post-progression survival than those with low *EPB41L4A-AS2* expression (*p* < 0.001and *p* = 0.0013, respectively; Figure 4A–4B). Subgroup analysis was also performed in lung cancer patients. As expected, lung cancer patients with high *EPB41L4A-AS2* expression exhibited better OS in various stratifications, such as chemotherapy status, tumor tissue histology, gender, and smoking history than patients with low *EPB41L4A-AS2* expression (all *p* < 0.05, Figure 4C–4D, Supplementary Figures S11–S16).

### *EPB41L4A-AS2* and its co-expressed genes play roles in tumor associated biological processes

**Figure 5:**
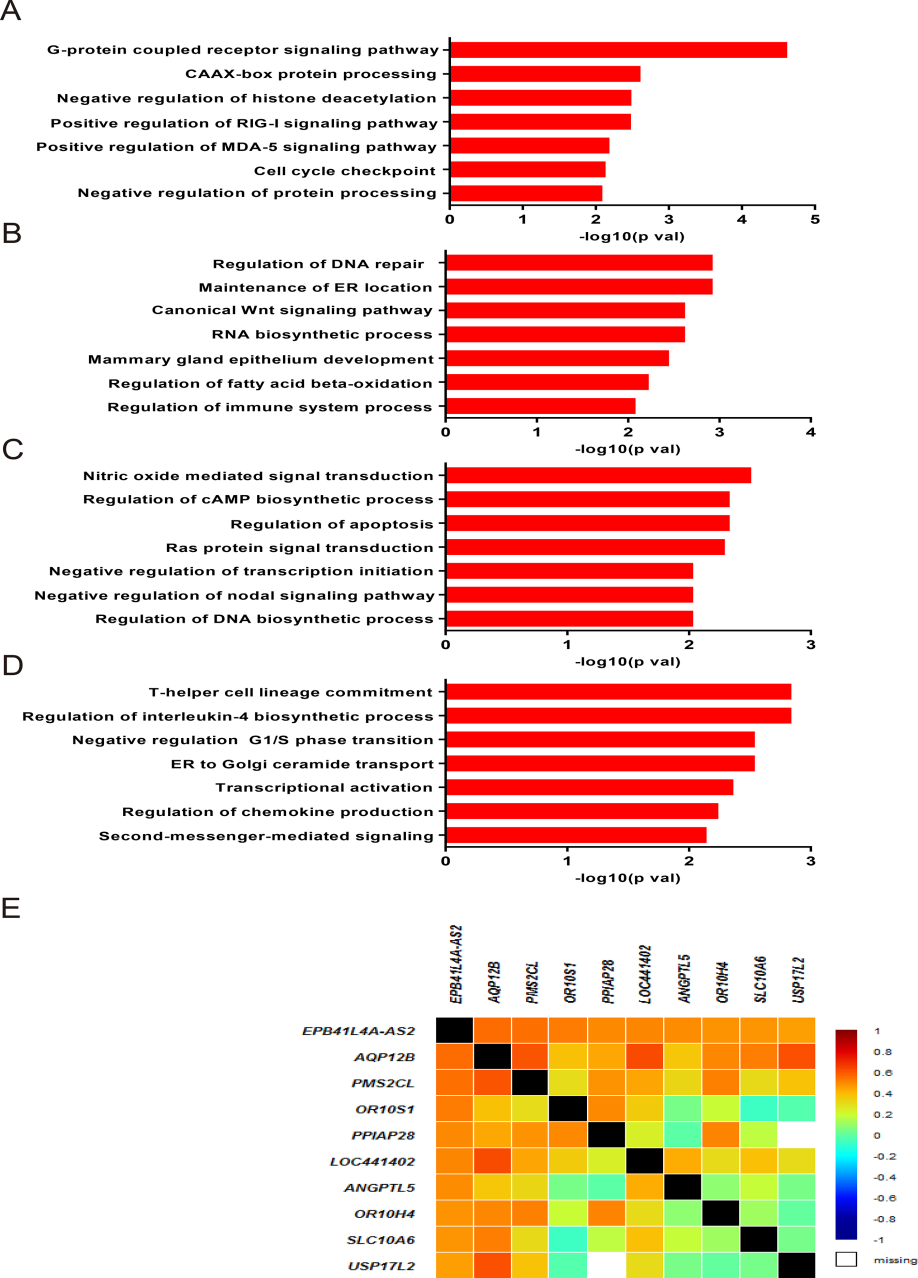
Associated biological processes of *EPB41L4A-AS2* co-expressed genes in breast cancer Gene ontology enrichment analysis for *EPB41L4A-AS2* co-expressed genes and correlated biological processes in breast cancer for all patients (**A**), in basal-like subtype (**B**), in luminal subtype (**C**) and in HER-2 subtype (**D**) in cohort IV. (**E**) Correlation between *EPB41L4A-AS2* and its representative coexpressed genes in cohort IV.

We speculated that *EPB41L4A-AS2* and its co-expressed genes could participate in various biological processes in tumor malignancies. Therefore, we investigated co-expressed genes and analyzed them with gene ontology (GO) enrichment analysis for breast cancer patients in cohort IV. *EPB41L4A-AS2* expression was correlated with tumor-associated biological processes, including G-protein coupled receptor signaling pathways, CAAX-box protein processing, negative regulation of histone deacetylation, the MDA-5 signaling pathway, the RIG-I signaling pathway, and cell cycle checkpoints (all *p* < 0.05; Figure 5A). We also performed the same analysis in breast cancer patients with different PAM50 molecular subtypes. Biological processes including DNA repair, maintenance of ER location, RNA biosynthesis, the canonical Wnt signaling pathway, mammary gland epithelial development, fatty acid beta-oxidation, and regulation of immune system processes were enriched in the basal-like subtype (all *p* < 0.05; Figure 5B). In the luminal subtype, the regulation of apoptosis, Ras signal transduction, endothelial cell migration, cell proliferation, and nodal signaling pathways were detected (all *p* < 0.05; Figure 5C). In the HER-2 subtype, biological processes involved in the regulation of cell cycle G1/S phase transition, second-messenger-mediated signaling, T-helper cell lineage commitment, interleukin-4 biosynthetic process, ER transport, transcriptional activation, and chemokine production were observed (all *p* < 0.05; Figure 5D). Representative co-expressed genes are shown in Figure 5E.

### Overexpression of *EPB41L4A-AS2* inhibits tumor cell proliferation

**Figure 6:**
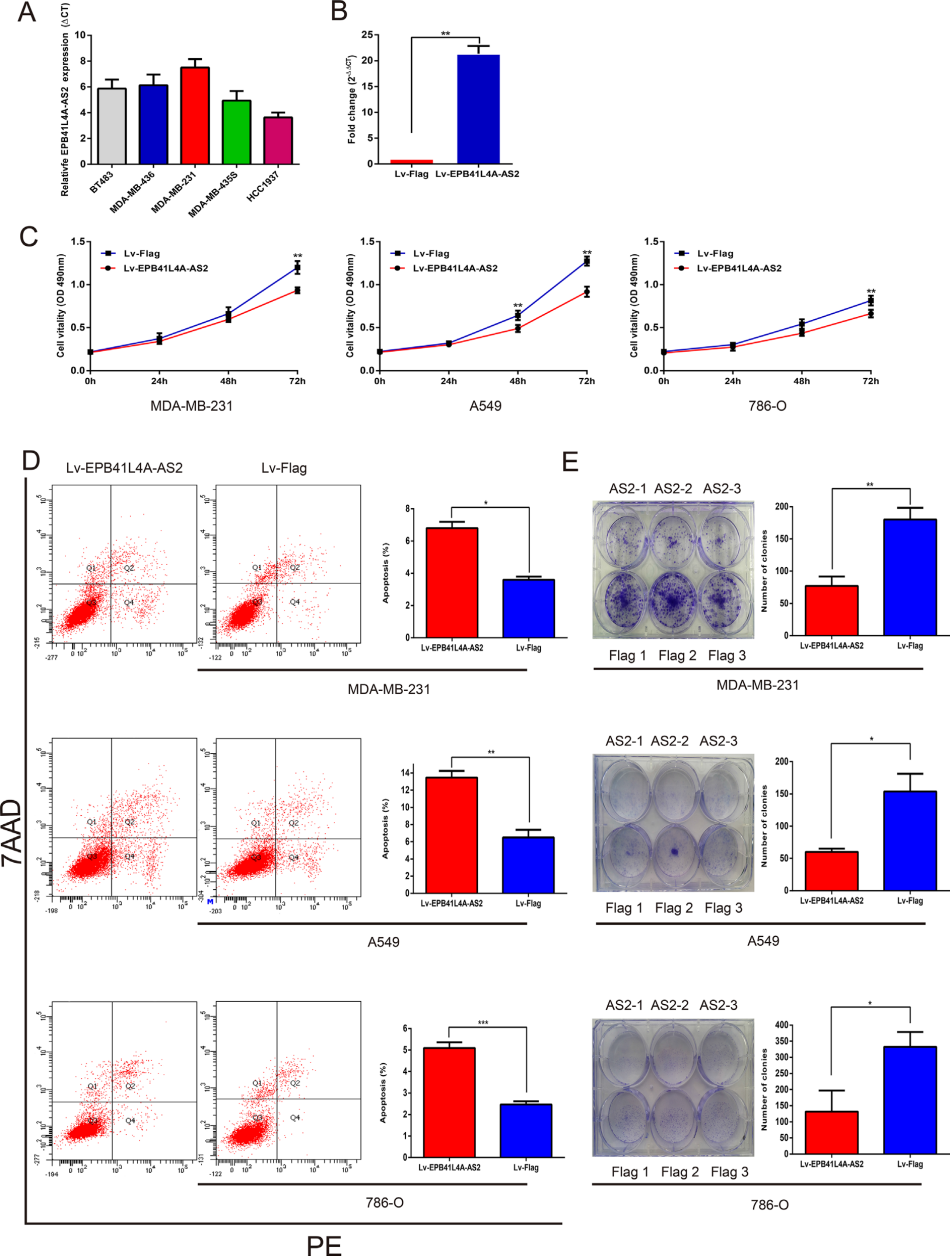
Ectopic overexpression of *EPB41L4A-AS2* inhibits tumor cell proliferation (**A**) Endogenous expression of *EPB41L4A-AS2* was detected in breast cancer cell lines. (**B**) Validation of ectopic overexpression of *EPB41L4A-AS2* by qRT-PCR. Ectopic overexpression of *EPB41L4A-AS2* inhibits proliferation (**C**), promotes apoptosis (**D**) and decreases colony formation (**E**) in MDA-MB-231, A549 and 786-O cell lines. qRT-PCR amplification was performed in triplicate and the levels of *EPB41L4A-AS2* were normalized against control *GAPDH* expression. Data are presented as the mean value from three independent experiments ± S.D. ***p < 0. 001; **p < 0. 01; *p < 0.05.

From the GO enrichment analysis, G-protein coupled receptor signaling pathways, histone deacetylation regulation, the RIG-I signaling pathway, and cell cycle checkpoint processes were found to be associated with tumor cell proliferation [39–46]. To elucidate the role of *EPB41L4A-AS2* in tumor cell proliferation, we overexpressed the lncRNA using purified lentivirus for *EPB41L4A-AS2* or a Flag control. First, we measured the endogenous expression of *EPB41L4A-AS2* in breast cancer cell lines (Figure 6A). We selected MDA-MB-231 cells for our *EPB41L4A-AS2* overexpression assay, and transfections were successful based on qRT-PCR analysis (Figure 6B). We also overexpressed *EPB41L4A-AS2* in the renal cancer cell line 786-O and the lung cancer cell line A549. Cell proliferation assays showed that overexpression of *EPB41L4A-AS2* inhibited tumor cell growth compared with the control in all three cancer cell lines (Figure 6C). Furthermore, all three cell lines exhibited increased apoptosis with *EPB41L4A-AS2* overexpression as assessed by flow cytometry (Figure 6D). Finally, in a clonogenic assay, overexpression of *EPB41L4A-AS2* reduced clone numbers in the three cancer cell lines (Figure 6E). These *in vitro* experiments suggest that upregulated *EPB41L4A-AS2* expression may suppress tumor cell proliferation, which is in agreement with our clinical findings.

## DISCUSSION

Recent analyses of the mammalian transcriptome have revealed that up to 70% of the genome is transcribed into lncRNAs, while only 2% of the genome serves as coding transcripts [47, 48]. Given the uncertainty regarding their roles in cancer and other biological processes, lncRNAs have been regarded as transcriptional noise in the genome [49, 50]. Despite the growing recognition of the importance of lncRNAs in tumor biology, it is still a challenge to scientists to determine the functions of the large number of lncRNAs.

Although aberrant expression of lncRNAs has been identified as a marker in various solid tumors, the expression of lncRNA *EPB41L4A-AS2* was unknown in malignant diseases. In this study, we demonstrated that *EPB41L4A-AS2* expression differed with tumor grade, tumor size, disease stage, receptor status, and molecular subtype in breast cancer. To further validate and confirm our initial result, we downloaded and analyzed data from TCGA. Since the lncRNA, *MALAT1*, was upregulated in solid tumor tissue compared with the corresponding normal tissue [51–53], we investigated whether *EPB41L4A-AS2* expression had a similar expression pattern in both renal cancer and lung cancer. Interestingly, low levels of *EPB41L4A-AS2* were observed in renal cancer and lung cancer tissues compared with normal tissues. The expression of *EPB41L4A-AS2* in high-staged tumors was also downregulated compared with those with low tumor stage in lung and renal cancers. Aberrant expression of *EPB41L4A-AS2* in tumor tissues may indicate that it serves as a tumor suppressor and that its downregulation contributes to tumorigenesis and disease progression.

Because lncRNAs can serve as prognostic markers for patients with malignancies, we next asked whether *EPB41L4A-AS2* expression could be a predictor of prognosis in solid tumors. We found that downregulation of *EPB41L4A-AS2* was associated with poor survival outcomes, suggesting potential role for this marker in the clinical management of breast cancer. More importantly, our findings were consistent across multiple cohorts and on the basis of more than 20 independent studies, which collectively included thousands of patients. The lncRNA *HOTAIR* was identified as a prognostic marker not only in breast cancer but also in other solid tumors, such as gastric cancer and lung cancer [54–59]. To determine whether *EPB41L4A-AS2* has similar prognostic potential we explored the relationship between its expression and patient prognosis in breast cancer, renal cancer, and lung cancer in another independent cohort. We consistently found that downregulation of *EPB41L4A-AS2* was associated with poor survival outcomes in three major cancers.

One classic approach to exploring the putative functions of lncRNAs is through a “guilt-by-association” method [60]. This method associates lncRNAs with biological processes based on a common expression pattern and identifies lncRNAs that are associated with specific cellular processes [61, 62]. Analysis of co-expressed lncRNA genes has revealed co-regulation of genes by lncRNAs, suggesting that lncRNA clusters may regulate biological processes. In this study, we examined genes co-expressed with *EPB41L4A-AS2* and performed GO enrichment analysis using cohort IV. Many tumor-associated biological processes, such as G-protein coupled receptor signaling pathways, negative regulation of cell cycle G1/S phase transition, and canonical Wnt signaling were identified. Using the guilt-by-association method, we propose that *EPB41L4A-AS2* may regulate tumor cell proliferation by mediating expression of some of these genes that are associated with tumor growth, but this association remains to be investigated in future studies.

Antisense lncRNA is transcribed from the opposite strand to a protein-coding gene [63, 64]. Antisense lncRNAs may act as epigenetic regulators to interfere with the transcription, or increase the mRNA stability, of coding genes [65, 66]. As a novel antisense lncRNA, it would be interesting to investigate whether *EPB41L4A-AS2* can mediate the transcription of the sense strand gene, *EPB41L4A*, or increase its mRNA stability. In this study, our *in vitro* experiments on breast cancer, renal cancer, and lung cancer cell lines demonstrated that overexpression of *EPB41L4A-AS2* inhibited tumor cell proliferation. These results were consistent with the findings of our clinical studies, which underscore the potential involvement of *EPB41L4A-AS2* in solid tumors. However, little is known about how and why *EPB41L4A-AS2* behaves like a tumor suppressor. Further studies are needed to determine how *EPB41L4A-AS2* is involved in mediating tumor biology.

## MATERIALS AND METHODS

### Patient specimens and clinical assessments

Eligible patients who had a histological diagnosis of breast cancer received neither chemotherapy nor radiotherapy before surgical resection. In total, 250 breast cancer tissues and 50 normal tissues were obtained from the Harbin Medical University Cancer Hospital in Harbin, China. All samples were frozen in liquid nitrogen immediately after surgical resections and only tumors with > 80% tumor cells were selected for RNA extraction. In clinic, breast cancer patients are classified into five main subtypes: luminal A, luminal B (HER2+), luminal B (HER2-), HER2 type, and triple negative based on the immunohistochemical tests for proteins ER, PR, HER2, and Ki-67 [67]. Two independent senior pathologists confirmed the pathological diagnosis and molecular subtype identification of each cancer tissue. This study conformed to the clinical research guidelines and was approved by the research ethics committee of the Harbin Medical University Cancer Hospital. We obtained written informed consent from all patients.

### RNA extraction and quantitative real-time PCR

Total RNA was extracted from fresh samples preserved and frozen in liquid nitrogen using TRIzol Reagent (Invitrogen, Carlsbad, CA, USA), according to the manufacturer's instructions. Total RNA (2 μg) was reverse transcribed into cDNA using High-Capacity cDNA Reverse Transcriptase Kits (Applied Biosystems, Foster City, USA). The relative level of *EPB41L4A-AS2* to the control gene *GAPDH* was determined by qRT-PCR using the 7500 Fast Real-Time PCR System (Applied Biosystems). The specific sequences of the primers used were 5′- CGGAGCAGGTGCAATCTGT-3′ (forward) and 5′-TCAAAACTACGTCTGATGCCAAA-3′ (reverse) for *EPB41L4A-AS2*; and 5′-ACCACAGTCCATGCCATCAC-3′ (forward) and 5′- TCCACCCTGTTGCTGTA-3′ (reverse) for *GAPDH*. PCR amplification was performed in triplicate, first at 95°C for 10 m, 40 cycles of 95°C for 10 s, and 60°C for 60 s. Quantitative normalization of *EPB41L4A-AS2* cDNA was performed in each sample using the expression of *GAPDH* as an internal control. Relative levels of *EPB41L4A-AS2* transcripts vs. *GAPDH* were determined by the comparative CT (2^−ΔCT^) method.

### Public data analysis

The genome-wide lncRNA expression profiles of breast cancer, renal cancer, and lung cancer were downloaded from TCGA (https://tcga-data.nci.nih.gov/). The expression of lncRNA *EPB41L4A-AS2* was calculated by summarizing the background corrected intensity of all probes mapped to this gene. Gene expression data for meta-analysis and prognosis analysis were selected from the GEO database. Meta-analysis included 24 datasets with 3699 breast cancer patients (Supplementary Table S1). Prognosis analysis included 26 datasets with 3554 breast cancer patients (Supplementary Table S2). The raw CEL files were downloaded from the GEO database (http://www.ncbi.nlm.nih.gov/geo/), and the background was adjusted according to a previously described method [68]. Expression of *EPB41L4A-AS2* was dichotomized using study-specific median expression as the cutoff to define “high value” at or above the median versus “low value” below the median. The meta-analysis was conducted with the use of Review Manager (Revman Version 5.3, Copenhagen, Denmark; http://tech.cochrane.org/revman/), whereas gene co-expression and gene ontology term enrichment were analyzed using DAVID as previously described [69, 70].

### Cell culture experiments

MDA-MB-231 cells were cultured in L-15 medium (Invitrogen, Carlsbad, CA) containing 10% fetal bovine serum and 100 units/ml of penicillin/streptomycin at 37°C without CO_2_. A549 and 786-O cells were cultured in a 1640 medium (Invitrogen, Carlsbad, CA) containing 10% fetal bovine serum and 100 units/ml of penicillin/streptomycin at 37°C in an atmosphere containing 5% CO_2_. All cell lines were obtained from the Chinese Type Culture Collection, Chinese Academy of Sciences. Cells were used in their logarithmic growth phase.

### Lentiviral construction and production

The full-length nucleotide sequence of *EPB41L4A-AS2* (NR_027706.1) was directly synthesized by Sangon Biotech Company and cloned into pLVX-Puro lentiviral expression vector (Clontech) between EcoRI and XbaI sites using an In-Fusion Cloning kit (Clontech). Lentiviruses were produced in 293T cells. Briefly, 293T cells were transiently transfected with pLVX plasmid and the packaging plasmids pLP1, pLP2, and pLP/VSVG using lipofectamine 2000. Lentiviral particles were harvested by collecting the supernatants 72 h later, which were centrifuged at 1500 g for 5 min and filtered through a 0.45 μm filter to remove cellular debris. Then, the crude lentivirus was concentrated by ultracentrifugation at 22,000 rpm for 2.5 h at 4°C, using a Beckman Ti70 rotor. The pellets were resuspended in DPBS and incubated at 4°C overnight. The purified lentivirus was then stored at −80°C.

### Cell proliferation assay

A cell proliferation assay was carried out using the Cell Counting Kit-8 according to the manufacturer's instructions (Beyotime, Shanghai, China). Briefly, 2 × 10^3^ cells were seeded in a 96-well plate. Cell proliferation was assessed for 24, 48, and 72 h. After adding 20 μl WST-1 reagents per well, cultures were incubated for 2 h and the absorbance was measured at 490 nm using a microplate reader (BioTek, VT, United States).

### Clonogenic assay

For the clonogenic assay, 500 cells per well were seeded in 6-well plates. Two weeks later, visible colonies were noticed using the naked eye, fixed with 4% formaldehyde, and stained with 0.1% crystal violet. The colonies with a diameter greater than 1 mm were counted.

### Flow cytometry

The Annexin-PE Apoptosis detection kit (BD Biosciences, San Jose, CA) was used to examine cell apoptosis according to the manufacturer's instructions. Briefly, cells were harvested by washing twice in cold PBS, and resuspending in 1 × Binding Buffer. Then, 100 μl of the cell solution (1 × 10^5^ cells) was transferred into a 5 ml culture tube, and 5 μl of Annexin V-PE and 5 μl of 7-AAD were added. The cells were gently vortexed and incubated for 15 min at RT (25°C) in the dark. Then, 400 μl of 1 × binding buffer was added to each tube, and apoptosis analysis was performed in a FACScan instrument (Becton Dickinson, Mountain View, CA, USA).

### Statistical analysis

Clinical and pathologic associations of *EPB41L4A-AS2* were determined using the Chi-square test. A Welch's unpaired *t* test was used to evaluate the difference of *EPB41L4A-AS2* expression among the PAM50 molecular subtypes. A significant *p*-value indicated that *EPB41L4A-AS2* expression was different in at least two subtypes. Kaplan–Meier method and log-rank test were performed to show survival differences according to *EPB41L4A-AS2* expression. The time for OS or RFS was calculated as the time from surgery until the occurrence of death and relapse, respectively. DMFS or PPS represented the time for distant metastasis-free survival and post progression survival, respectively. AE represented any event including metastasis, relapse, or death. The differences between groups in our *in vitro* experiments were analyzed using a Student's *t*-test. Spearman correlation coefficients were calculated for correlation analysis. All experiments were performed in triplicate, and the SPSS 16.0 software system (SPSS, Chicago, IL) was used for statistical analysis. All statistical tests were two-sided, and *p* < 0.05 was considered statistically significant.

## SUPPLEMENTARY MATERIALS FIGURES AND TABLES


