# Supplementary material for: The long non-coding RNA EPB41L4A-AS2 inhibits tumor proliferation and is associated with favorable prognoses in breast cancer and other solid tumors
Source: Oncotarget. 2016 Mar 9;7(15):20704–17. doi: 10.18632/oncotarget.8007 (PMC4991486; doi:10.18632/oncotarget.8007)
Supplement: Supplementary file 1 [file oncotarget-07-20704-s001.pdf]

The long non-coding RNA *EPB41L4A-AS2* inhibits tumor proliferation and is associated with favorable prognoses in breast cancer and other solid tumors

Supplementary Materials

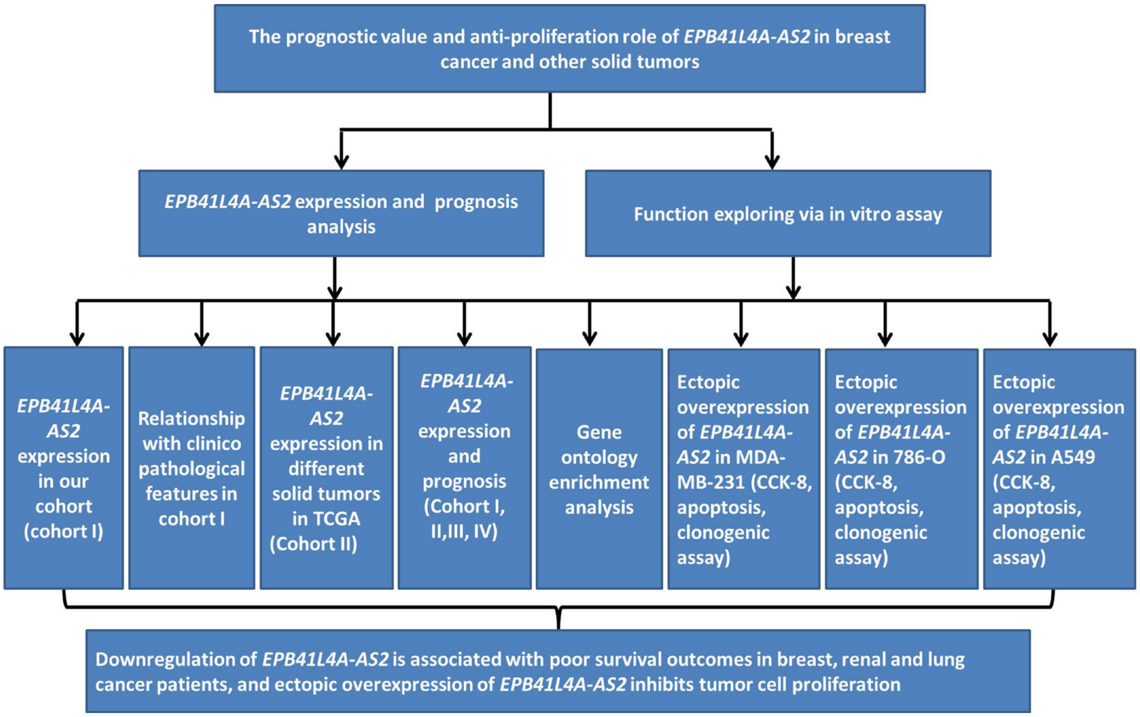

Supplementary Figure S1: Flow chart of this study.

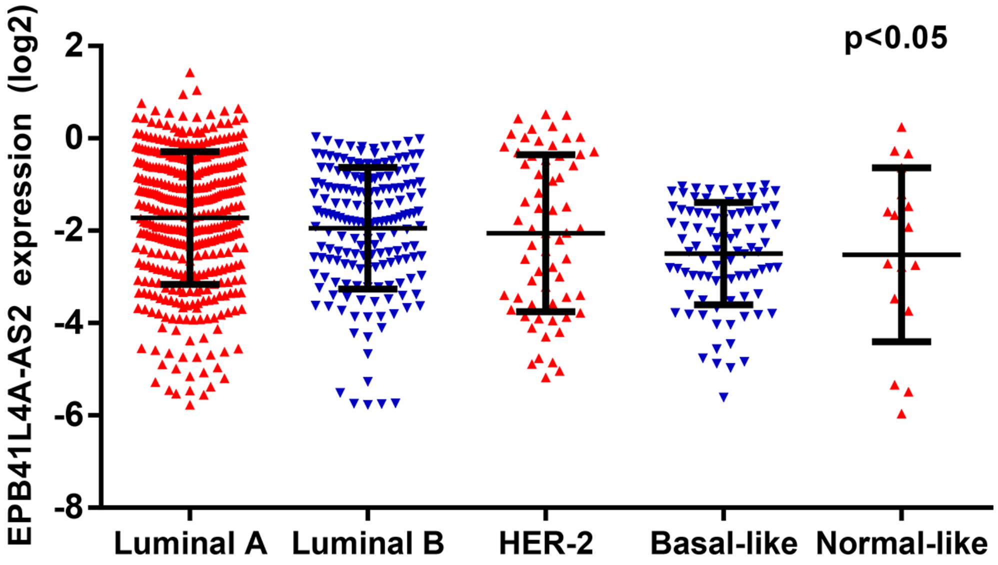

Supplementary Figure S2: *EPB41L4A-AS2* expression in five different molecular subtype breast cancer tissues.

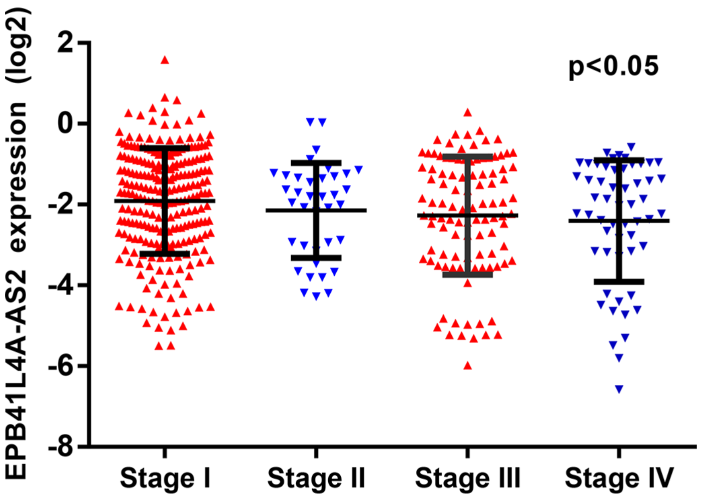

Supplementary Figure S3: ***EPB41L4A-AS2*** **expression in renal cancer with different stage.**

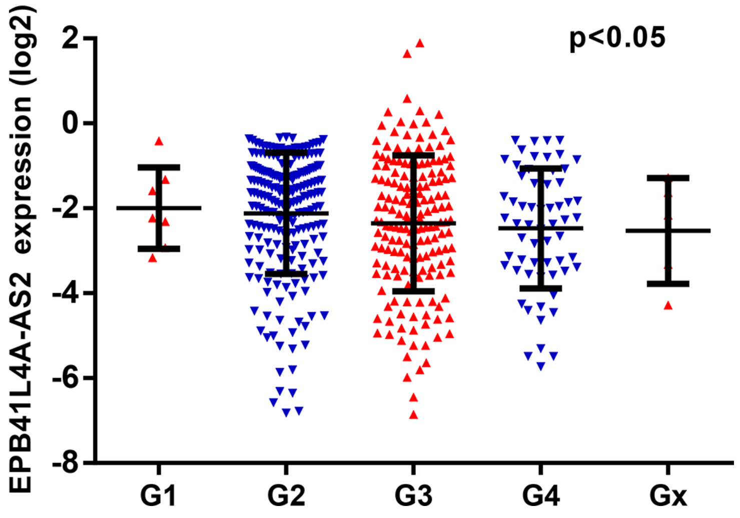

Supplementary Figure S4: ***EPB41L4A-AS2*** **expression in renal cancer with different grade.**

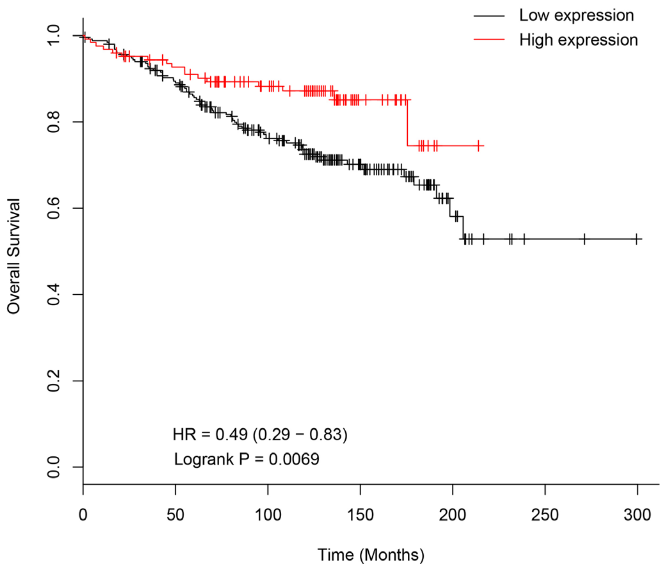

Supplementary Figure S5: High ***EPB41L4A-AS2*** **expression was associated with better OS than low expression in patients with ER positive breast cancer in cohort III (*N* = 377).**

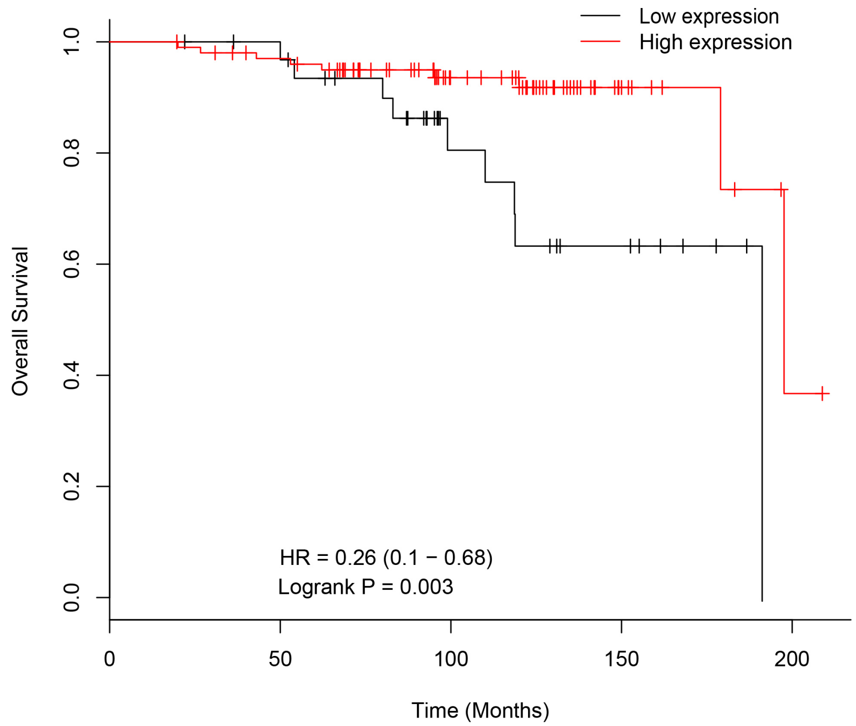

Supplementary Figure S6: High *EPB41L4A-AS2* expression was associated with better OS than low expression in patients with low tumor grade in cohort III (*N* = 135).

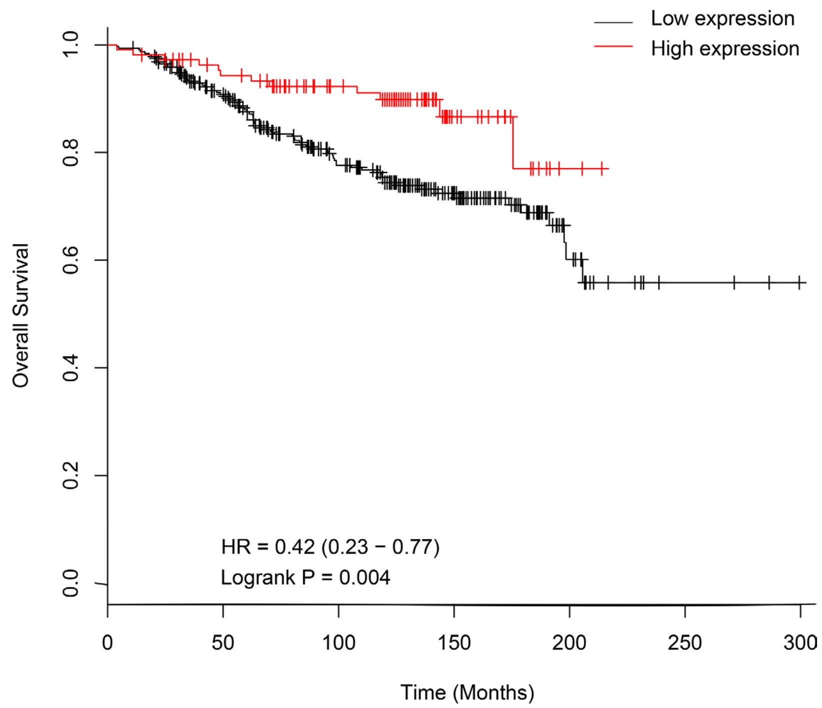

Supplementary Figure S7: High *EPB41L4A-AS2* expression was associated with better OS than low expression in patients with uninvolved lymph nodes in cohort III (*N* = 425).

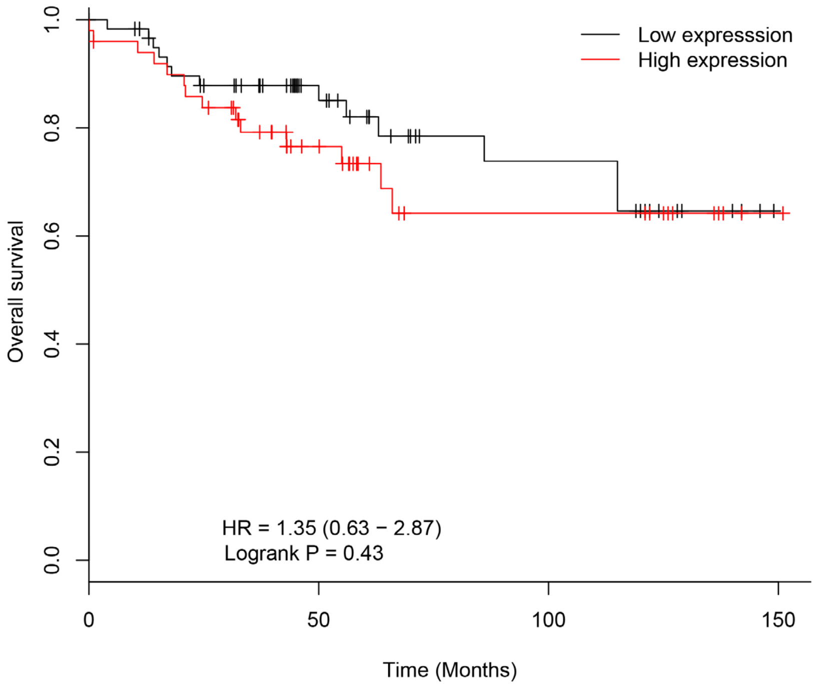

Supplementary Figure S8: High *EPB41L4A-AS2* expression was associated with worse OS than low expression in breast cancer patients with a *TP53* mutation (N = 111).

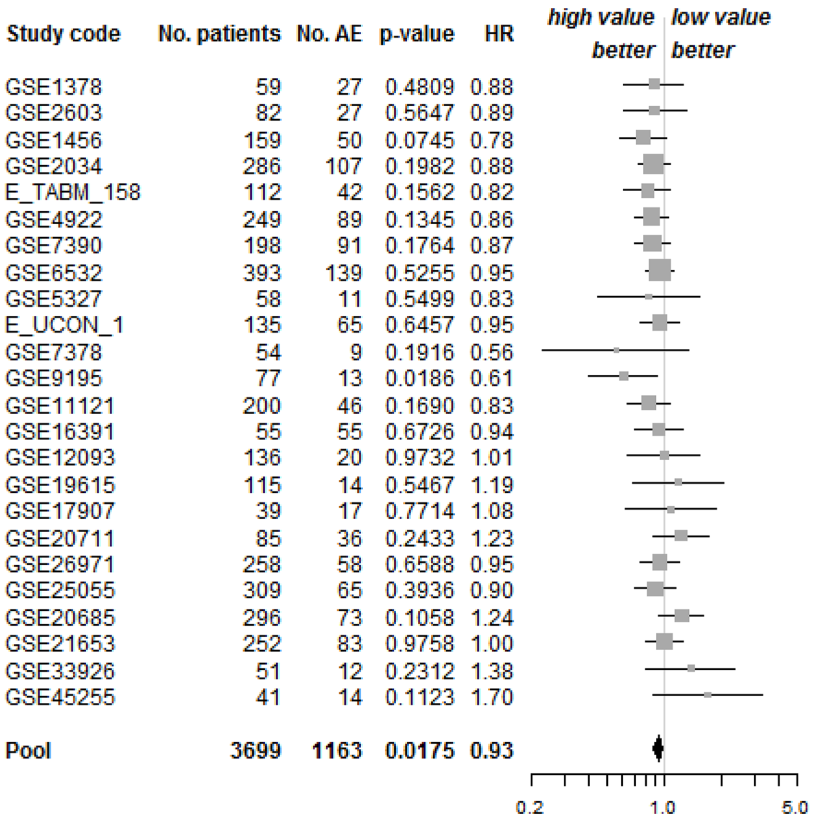

Supplementary Figure S9: Meta-analysis of *EPB41L4A-AS2* expression and prognosis of any event in breast cancer patients in cohort IV (N = 3,699).

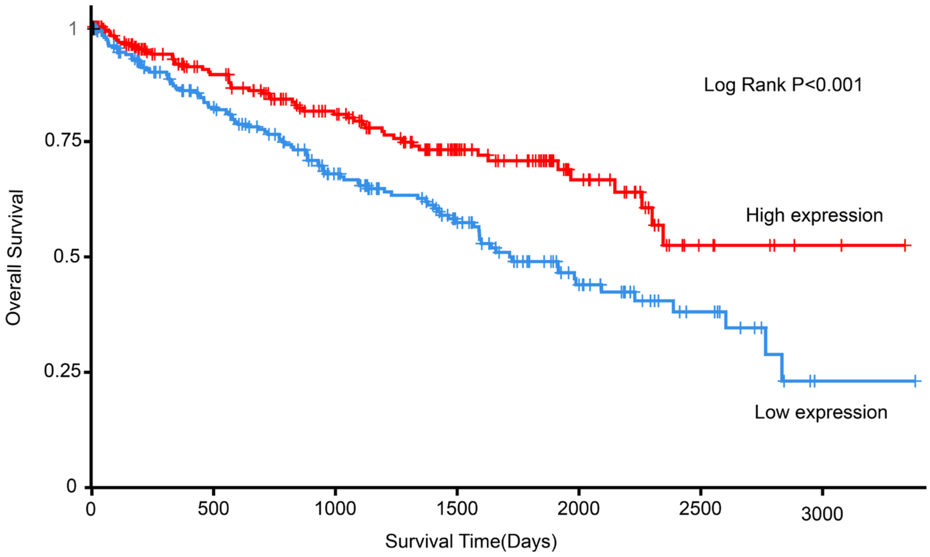

Supplementary Figure S10: High *EPB41L4A-AS2* expression was associated with better OS than low expression in renal cancer patients in cohort II (*N* = 448).

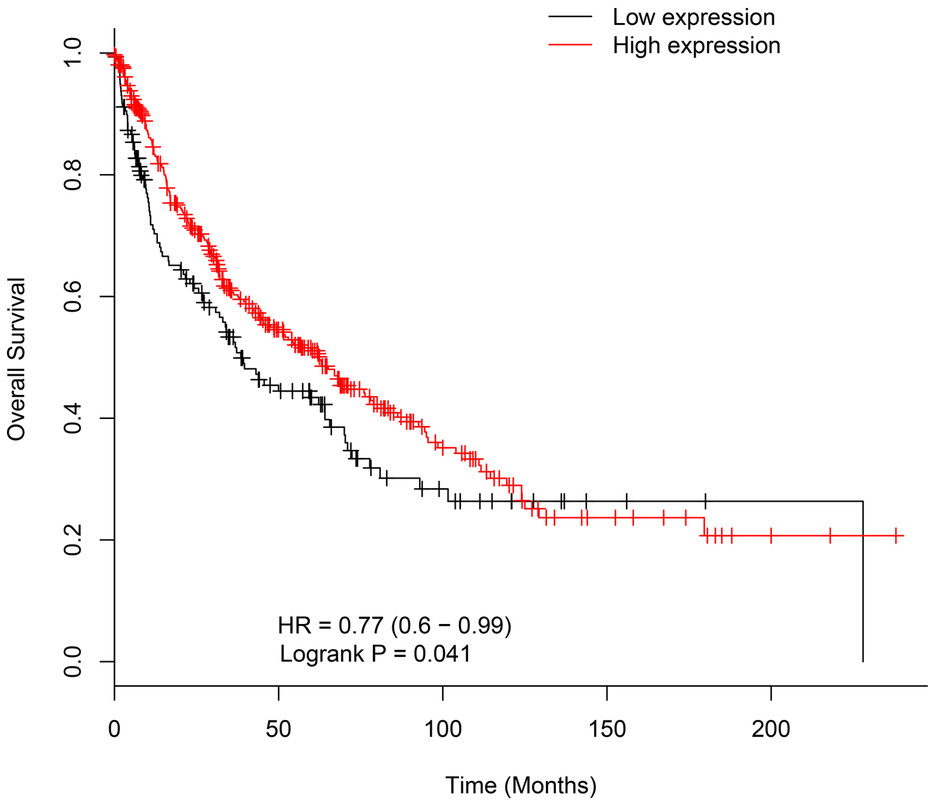

Supplementary Figure S11: High *EPB41L4A-AS2* expression was associated with better OS than low expression in lung squamous cell cancer patients (*N* = 524).

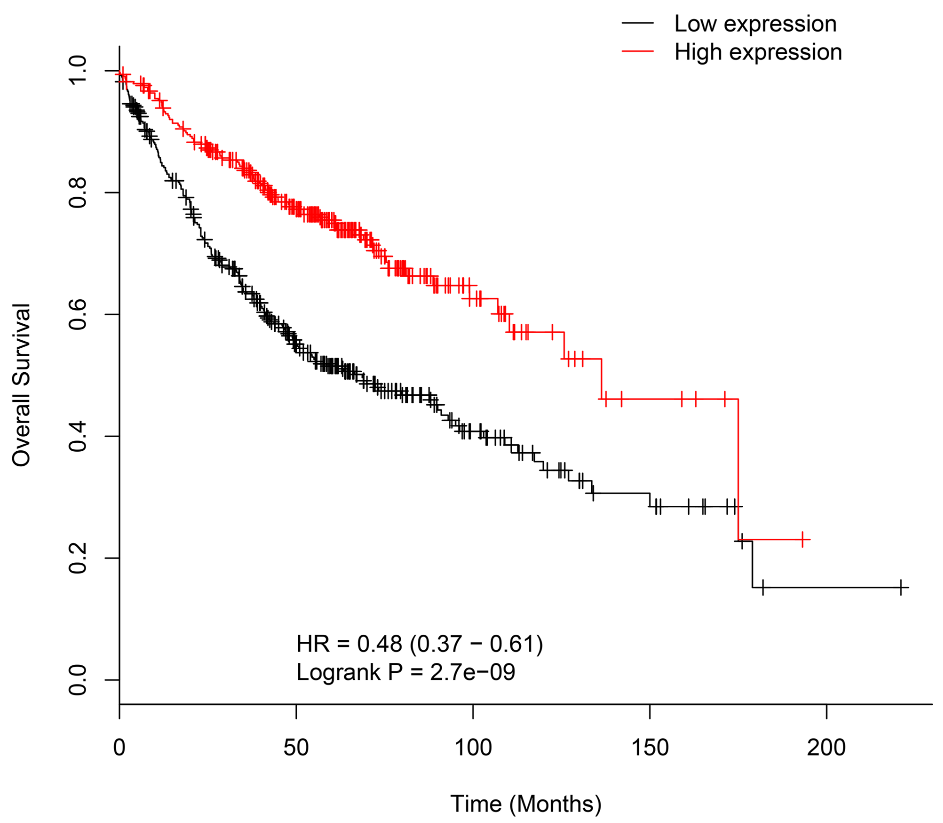

Supplementary Figure S12: High *EPB41L4A-AS2* expression was associated with better OS than low expression in lung adenocarcinoma patients (*N* = 720).

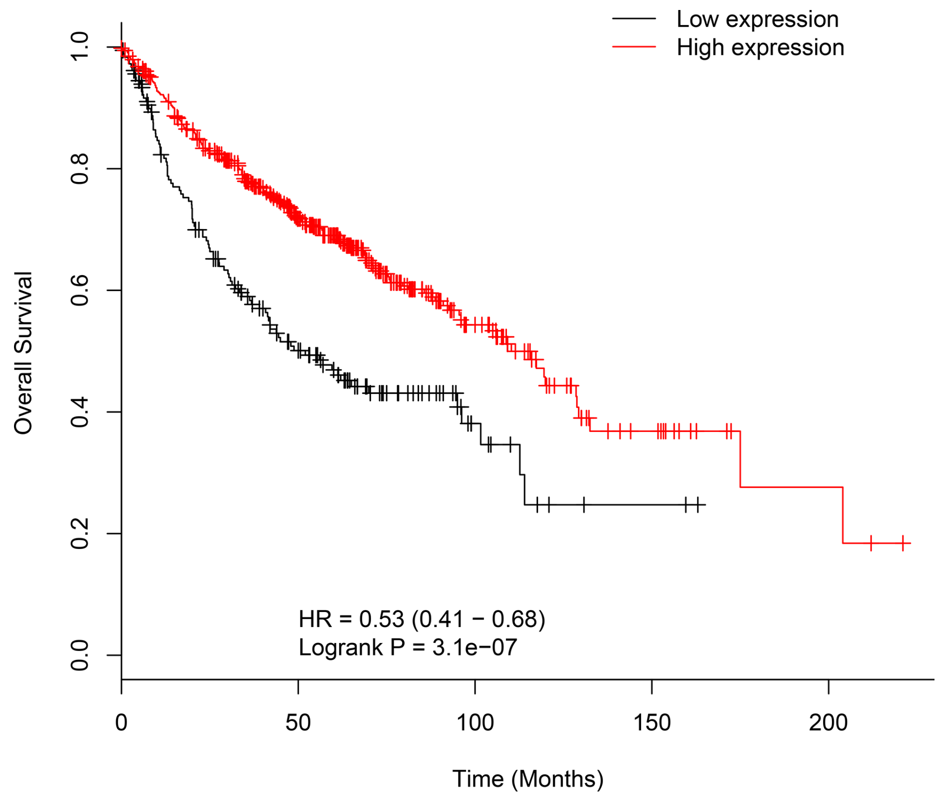

Supplementary Figure S13: High *EPB41L4A-AS2* expression was associated with better OS than low expression in female lung cancer patients (*N* = 715).

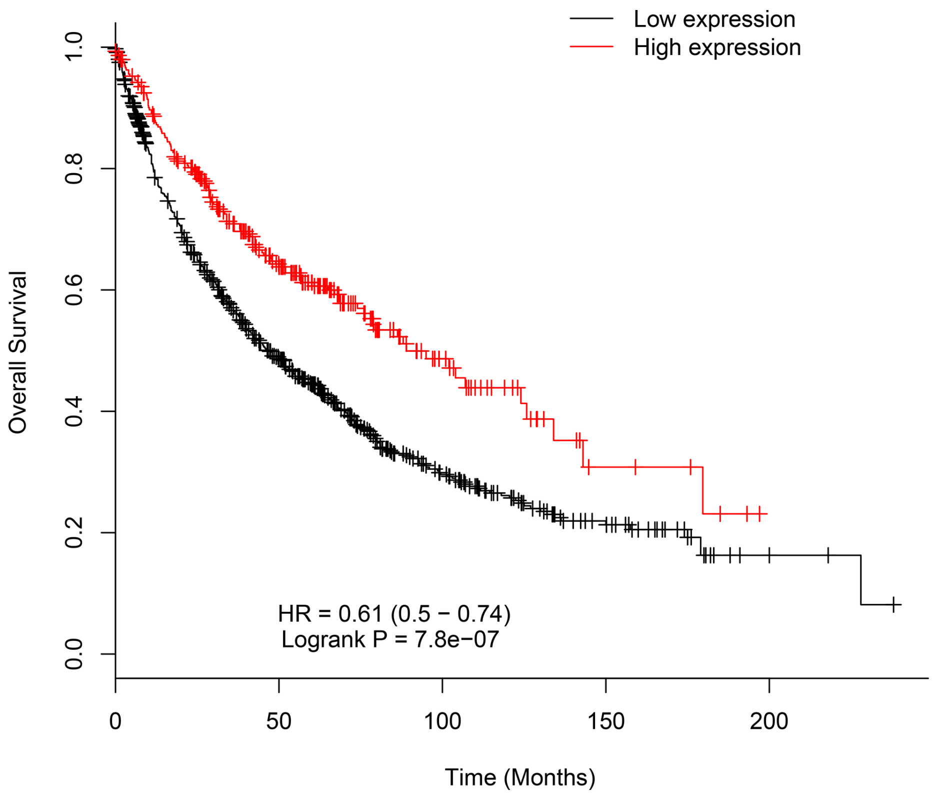

Supplementary Figure S14: High *EPB41L4A-AS2* expression was associated with better OS than low expression in male lung cancer patients (*N* = 1,100).

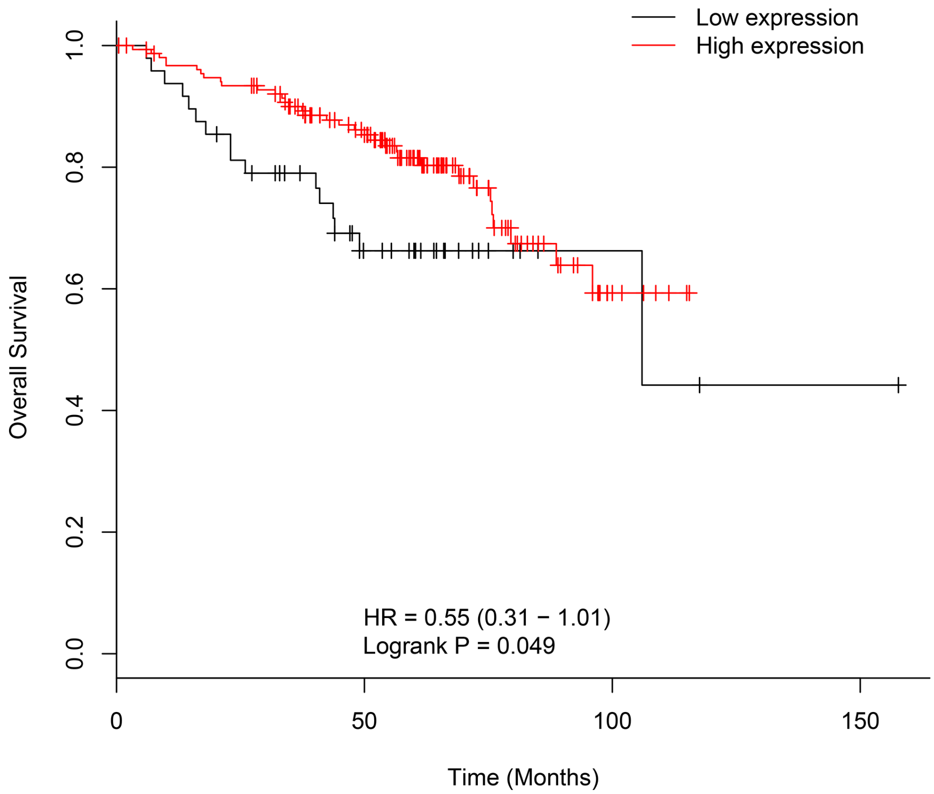

Supplementary Figure S15: High *EPB41L4A-AS2* expression was associated with better OS than low expression in lung cancer patients who never smoked (*N* = 205).

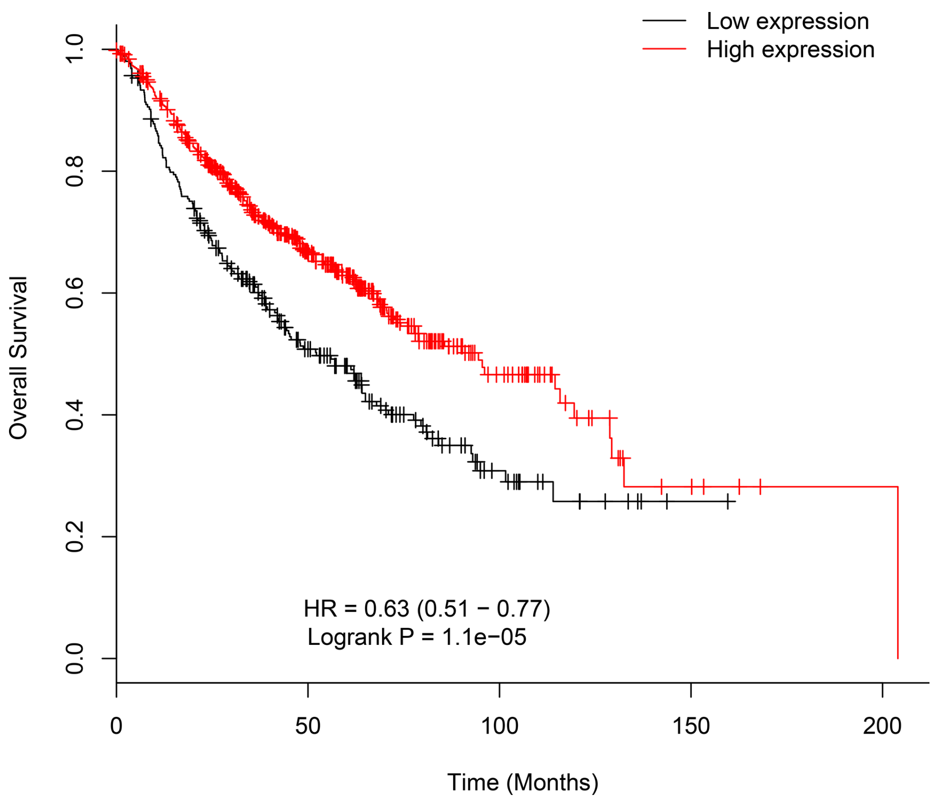

Supplementary Figure S16: High *EPB41L4A-AS2* expression was associated with better OS than low expression in lung cancer patients who smoked (*N* = 820). $^{****}p < 0.0001$; $^{***}p < 0.001$; $^{**}p < 0.01$; $^{*}p < 0.05$.

Supplementary Table S1: GEO datasets list in cohort III

Cohort III
GSE12276
GSE11121
GSE12093
GSE1456
GSE16391
GSE16446
GSE17907
GSE17705
GSE19615
GSE20194
GSE20271
GSE2034
GSE20685
GSE20711
GSE21653
GSE25055
GSE26971
GSE26990
GSE31448
GSE31519
GSE3494
GSE5327
GSE6532
GSE7390
GSE9195
E_MTAB_365

Supplementary Table S2: GEO datasets list in cohort IV

Cohort IV
GSE1378
GSE11121
GSE12093
GSE1456
GSE16391
GSE17907
GSE19615
GSE2034
GSE20685
GSE20711
GSE21653
GSE25055
GSE26971
GSE2603
GSE33926
GSE45255
GSE4922
GSE5327
GSE6532
GSE7390
GSE7378
GSE9195
E_TABM_158
E_UCON_1
